# Processing Parameter Effects on Residual Stress and Mechanical Properties of Selective Laser Melted Ti6Al4V

**DOI:** 10.1007/s11665-018-3477-5

**Published:** 2018-07-17

**Authors:** Haider Ali, Hassan Ghadbeigi, Kamran Mumtaz

**Affiliations:** Department of Mechanical Engineering, University of Sheffield, Sheffield, UK

**Keywords:** additive manufacturing, cooling rate, finite element, residual stress, layer thickness, mechanical properties, selective laser melting, Ti6Al4V

## Abstract

Selective laser melting (SLM) process is characterized by large temperature gradients resulting in high levels of residual stress within the additively manufactured metallic structure. SLM-processed Ti6Al4V yields a martensitic microstructure due to the rapid solidification and results in a ductility generally lower than a hot working equivalent. Post-process heat treatments can be applied to SLM components to remove in-built residual stress and improve ductility. Residual stress buildup and the mechanical properties of SLM parts can be controlled by varying the SLM process parameters. This investigation studies the effect of layer thickness on residual stress and mechanical properties of SLM Ti6Al4V parts. This is the first-of-its kind study on the effect of varying power and exposure in conjunction with keeping the energy density constant on residual stress and mechanical properties of SLM Ti6Al4V components. It was found that decreasing power and increasing exposure for the same energy density lowered the residual stress and improved the % elongation of SLM Ti6Al4V parts. Increasing layer thickness resulted in lowering the residual stress at the detriment of mechanical properties. The study is based on detailed experimental analysis along with finite element simulation of the process using ABAQUS to understand the underlying physics of the process.

## Introduction

Considerable research has focused on the effect of in-process parameters on residual stress buildup in SLM components (Ref 1-22). SLM process can be approximated by stacking of thousands of welds together; therefore, it is really important to understand the dynamics of a single weld or in the terminology of SLM a single melt pool. Melt-pool size increases with increasing energy input (Ref 23). Laser power has a more pronounced effect on the maximum temperature than exposure time (Ref 23). The maximum power depends on the laser hardware. Lowering the laser power reduces the maximum temperature of a melt pool (Ref 23-25) and also leads to a smaller melt pool, which results in higher cooling rates (Ref 24). High laser power results in lower deformation due to residual stress (Ref 11), while Alimardani et al. (Ref 25) reported lower residual stresses for lower laser power.

The effect of scan speed is the opposite of power. Reducing scan speed leads to lower temperature gradients (Ref 7), lower cooling rates (Ref 24), lower residual stresses (Ref 26) and reduced deformation, while higher scan speeds produce increased cooling rate and leads to increased cracking (Ref 27). Pohl et al. (Ref 10) reported lower deformation for higher scan speed. Combined effect of varying power and exposure together keeping energy density constant on porosity and in turn on mechanical properties was studied by Andrei et al. (Ref 28). For a constant energy density, lower power and higher exposure combination led to an increase in porosity and thus reduction in yield strength of 316L SLM samples (Ref 28).

To date no prior study has reported the effect of constant energy density with varying power and exposure together on residual stress and mechanical properties in SLM Ti6Al4V parts. This study investigates the effect of varying power and exposure while keeping energy density constant on residual stress and mechanical properties of SLM Ti6Al4V components.

Powder particle size determines the lower limit of the layer thickness, while the need for melt-pool penetration into underlying layers determines the upper limit. Larger layer thicknesses can increase productivity at the detriment of geometrical resolution, as well as roughness of side surfaces. It has been reported (Ref 12, 14, 18) that increasing layer thickness results in reduced residual stresses due to the reduction in cooling rate. According to Kruth et al. (Ref 18), for the same energy density doubling the layer thickness reduced the curling angle of a bridge geometry by 6%. According to Roberts et al. (Ref 17), doubling the layer thickness reduced the residual stress by 5%. According to Zaeh et al. (Ref 14), increasing the layer thickness by 2.5 times decreased the deformation of the ends of a T-shaped cantilever by 82%. Sufiiarov et al. (Ref 29) reported an increase in yield strength and a decrease in elongation for decreasing layer thickness in IN718 SLM parts. Guan et al. (Ref 30) reported that layer thickness had no effect on the mechanical properties of 304 stainless steel SLM components. Delgado et al. (Ref 31) reported that increasing layer thickness had a negative effect on the mechanical properties of AISI 316L SLM components. Parts were created with different layer thicknesses using the same parameters (Ref 12, 14, 17, 18, 29-31), optimized for one layer thickness. From the published work, the effect of layer thickness on residual stress and mechanical properties is not well understood.

This work presents a comprehensive study on the effect of varying power and exposure in conjunction with maintaining energy density constant on residual stress buildup and mechanical properties of SLM Ti6Al4V parts. This work studies the effect of layer thickness on residual stress and mechanical properties by individually optimizing the process parameters for each layer thickness. FEA simulation is used in combination with experimental trials to understand the underlying phenomena associated with the residual stress buildup and the trend in mechanical properties of SLM Ti6Al4V samples.

## Experimental Methodology

### Material and Processing Parameters

The composition of Ti6Al4V-ELI powder from Technik Spezialpulver (TLS) used in this work can be found in the work by Ali et al. (Ref 21). This work was carried out on the Renishaw AM250 machine using the process parameters presented in Table 1.

**Table 1:** SLM process parameters

Focus offset	Hatch spacing, µm	Contour spacing, mm	Point distance, µm	Scanning strategy
0	80	0.2	65	90° alternate

### Density and Microstructure

Density and microstructure for all test cases shown in Table 2 and 3 were analyzed based on the methodology presented in the work by Ali et al. (Ref 21).

**Table 2:** Constant energy density test cases

Test case	S-1	CED-1	CED-2	CED-3	CED-4
Power, W and exposure, µs	200 and 100	180 and 111	170 and 118	160 and 125	150 and 133

**Table 3:** Layer thickness test cases

Test case	LT-1	LT-2	LT-3
Layer thickness, µm	25	50	75
Power, W	170	200	200
Exposure, µs	80	100	120

### Mechanical Properties and Residual Stress

**Fig. 1:**
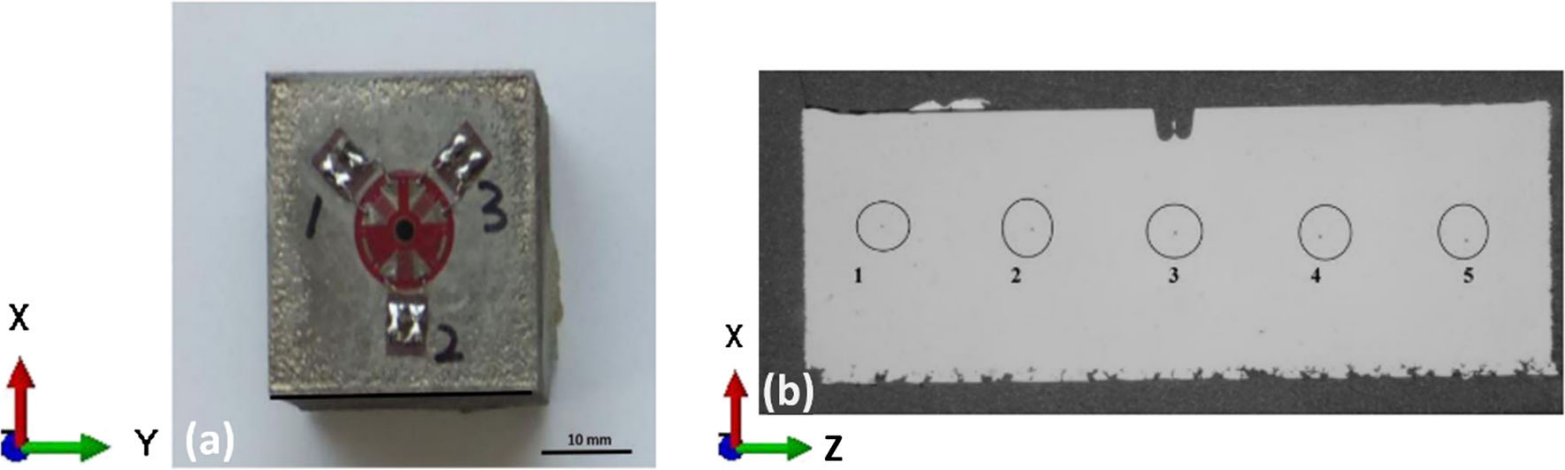
(a) 30 × 30 × 10 residual stress block showing hole drilled in the center of the strain gage rosette attached on top surface. (b) Cross-sectioned 30 × 30 × 10 residual stress block showing the locations of the Vickers hardness indentations

Three tensile test specimens and three 30 × 30 × 10 mm residual stress measurement blocks shown in Fig. 1(a) with strain gage attached to top surface were manufactured for each test case. Residual stress, mechanical properties and hardness (indentation locations shown in Fig. 1b) were tested based on the methodology presented in the work by Ali et al. (Ref 21).

### Varying Power and Exposure Combinations at Constant Energy Density

Test specimens were manufactured using 50 µm layer thickness for all test cases shown in Table 2. Power and exposure time were varied, such that the energy density for each build remained constant at 76.92 $\frac{\text{J}}{{{\text{mm}}^{ 3} }}$ as calculated from the optimum combination of parameters for 50-µm-layer-thickness density optimization trials presented in the work by Ali et al. (Ref 22). using ${\text{ED}} = \frac{P \times t}{{{\text{pd}} \times h \times {\text{lt}}}}$. where *P* is power in watts, *t* is exposure in μs, pd is point distance in μm, *h* is hatch spacing in μm, and lt is layer thickness in μm.

### Layer Thickness

Table 3 shows different layer thickness test cases with optimum power and exposure (determined from density optimization trials for each layer thickness) used for producing test specimens.

### Finite Element Simulation

The melting behavior of a single line containing 14 laser spots assigning powder properties to the top layer and solid properties to the substrate was simulated for all the cases shown in Tables 2 and 3. The modeling approach used within this work is based upon the work by Ali et al. (Ref 21).

## Results and Discussion

### Effect of Varying Power and Exposure with Constant Energy Density on Melt-Pool Size and Cooling Rate

FEA simulation with different combinations of power and exposure was used to estimate equivalent melt pool and cooling rates.

**Fig. 2:**
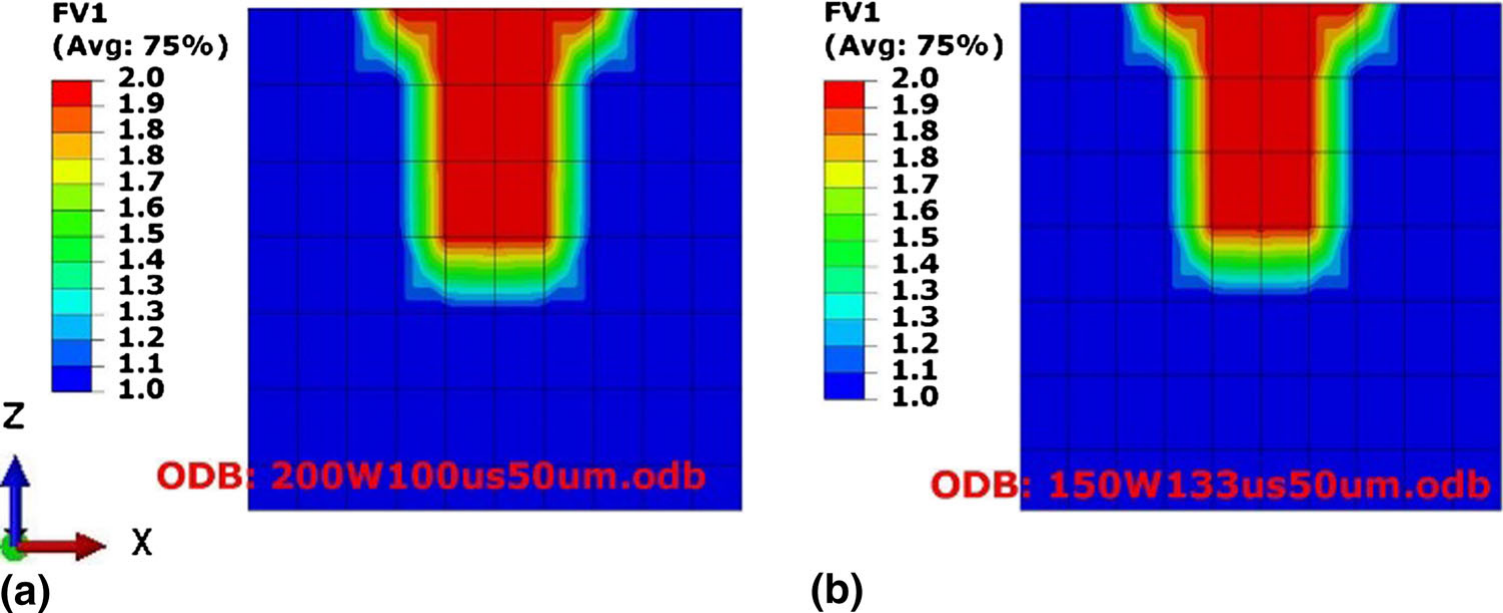
Effect of varying power and exposure time combination for a constant energy density on melt-pool dimensions. (a) Test case S-1 (200 W power and 100 µs exposure). (b) Test case CED-4 (150 W power and 133 µs exposure)

Figure 2(a) shows melt-pool dimensions for 200 W and 100 µs, while Fig. 2(b) shows CED-4 manufactured with the lowest power of 150 W and highest exposure of 133 µs tested in this work. Figure 2 shows that for constant energy density any combination of power and exposure resulted in the same melt-pool size.

**Fig. 3:**
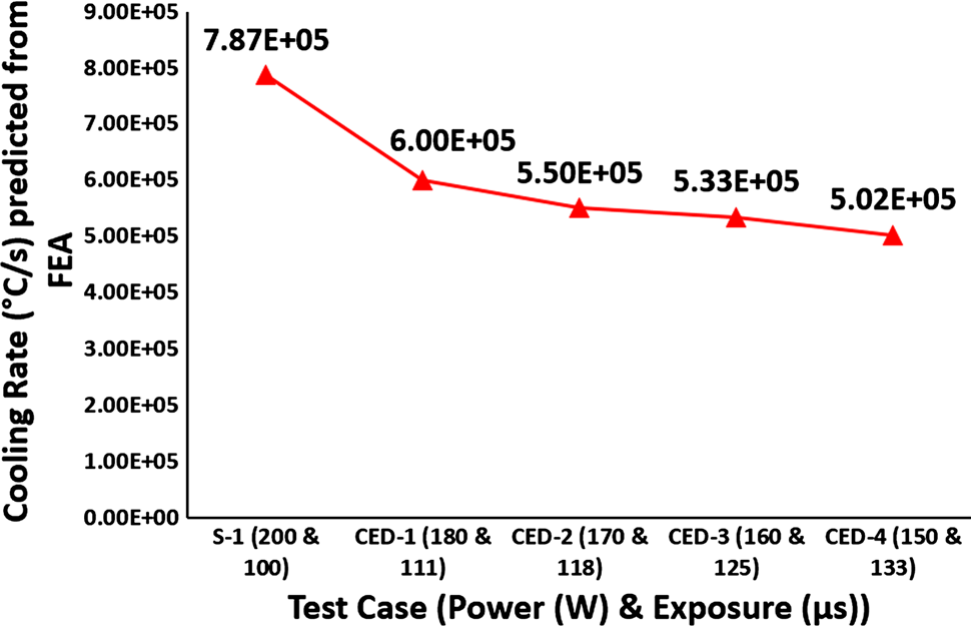
Effect of varying power and exposure time combination for a constant energy density on cooling rates

**Fig. 4:**
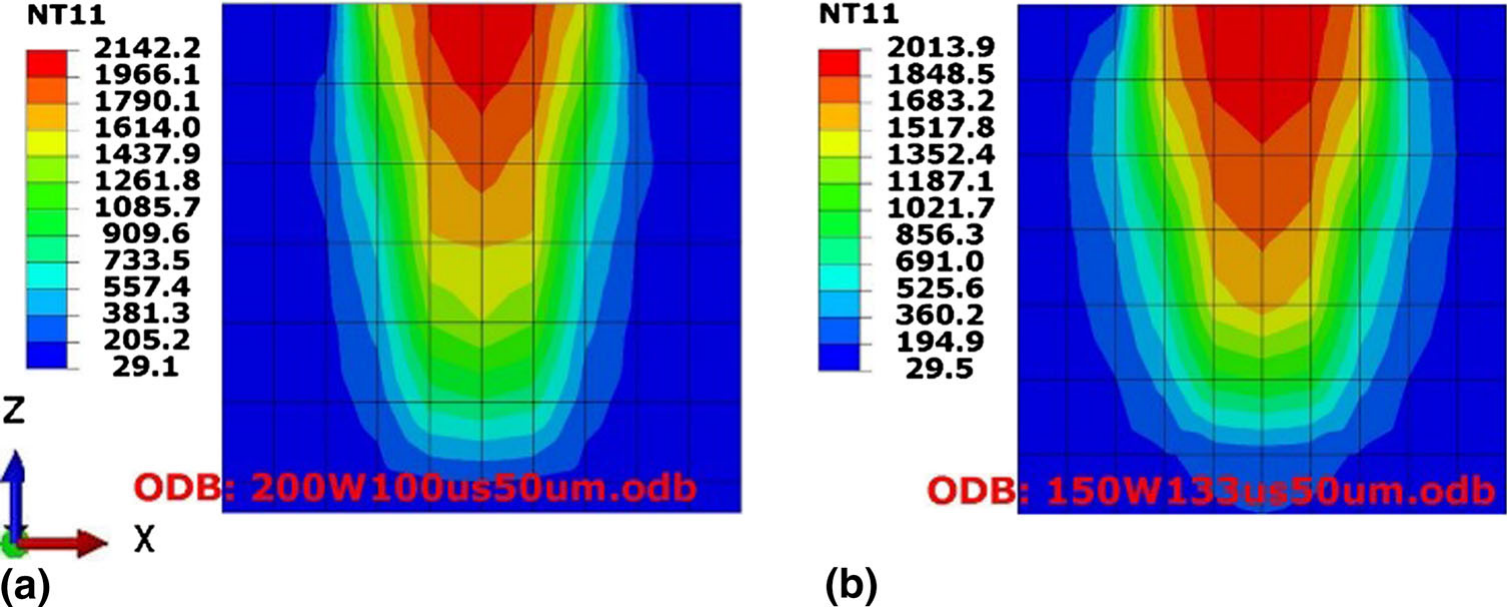
Effect of varying power and exposure time combination for a constant energy density on melt-pool temperature distribution. (a) Test case S-1 (200 W power and 100 µs exposure). (b) Test case CED-4 (150 W power and 133 µs exposure)

Figure 3 shows that for constant energy density, decreasing power and increasing exposure leads to a decrease in cooling rate. Figure 4 shows the temperature distribution across the depth of the melt pool, where Fig. 4(b) shows that adding the same energy at a slower rate provides time for heat flow to the surrounding material and raises its temperature. This heating of the surrounding material is responsible for the decrease in cooling rates depicted in Fig. 3.

#### Effect of Laser Power and Exposure with Constant Energy Density on Porosity and Microstructure

**Fig. 5:**
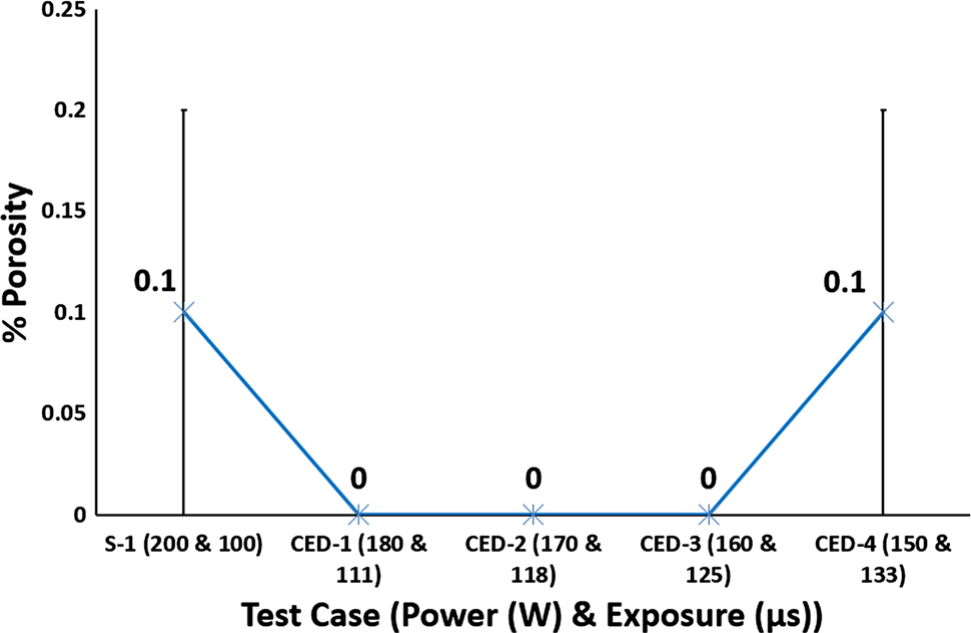
Effect of varying power and exposure time combination for a constant energy density on % porosity

Figure 5 shows that for a constant energy density all test cases achieved nearly fully dense SLM Ti6Al4V parts.

No variation in % porosity is consistent with the findings of Fig. 2 showing same melt-pool dimensions for all test cases.

**Fig. 6:**
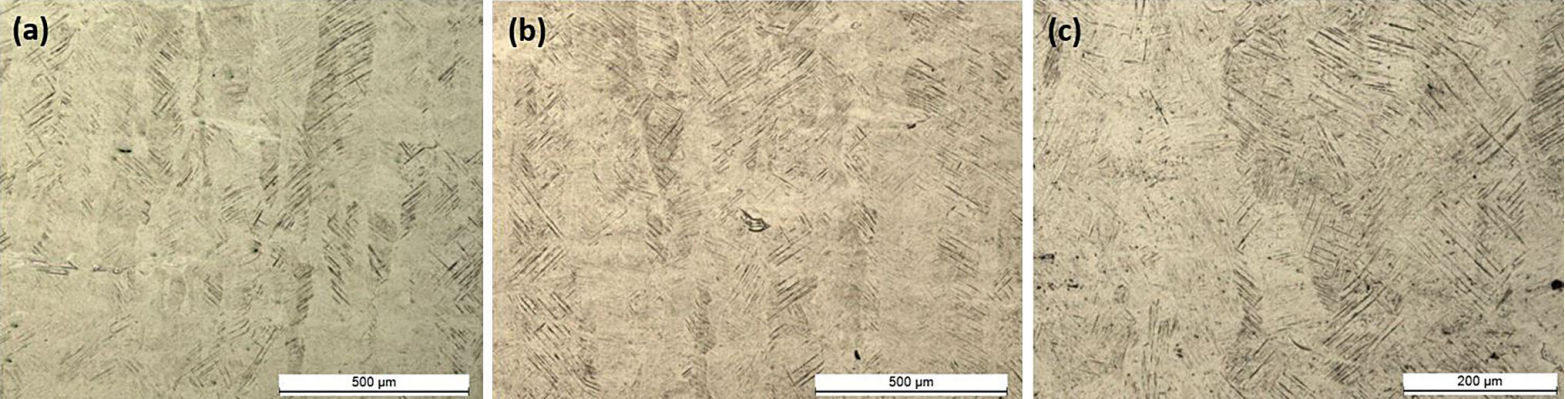
Martensitic α′ laths in prior β columnar grains. (a) Test case S-1, 90° alternating scanning strategy with optimum combination of power (200 W) and exposure (100 µs). (b) Test case CED-1, power (180 W) and exposure (111 µs). (c) Test case CED-4, power (150 W) and exposure (133 µs)

According to Ahmed et al. (Ref 32), cooling rates higher than 410 $\frac{{{^\circ }{\text{C}}}}{\text{s}}$ lead to fully martensitic microstructure for Ti6Al4V. Therefore, according to the cooling rates shown in Fig. 3, irrespective of the power and exposure combinations all test cases resulted in fully martensitic microstructure with martensitic $\alpha^{\prime}$ laths growing inside prior β columnar grains shown in Fig. 6.

#### Effect of Laser Power and Exposure with Constant Energy Density on Residual Stress

**Fig. 7:**
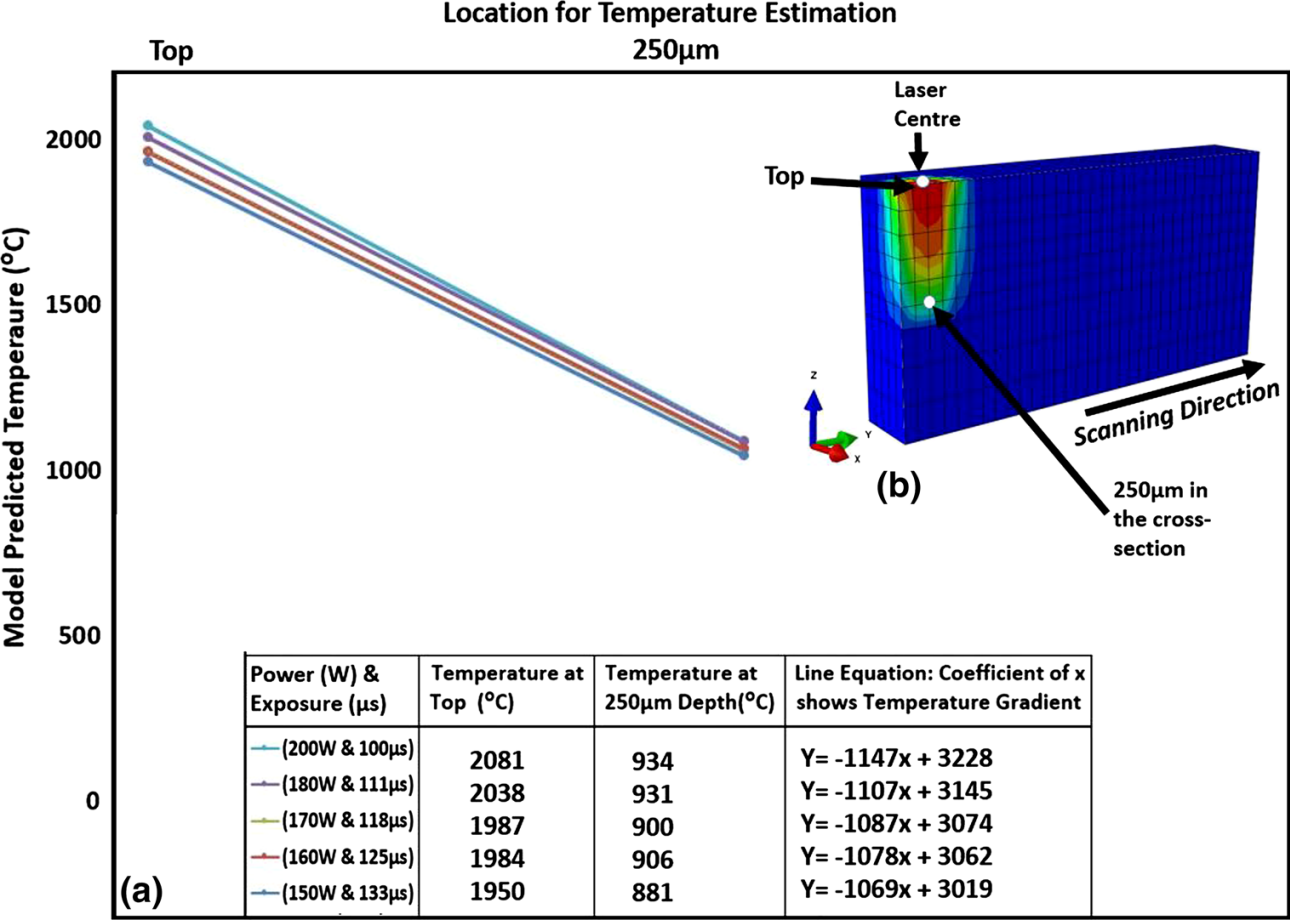
Effect of varying power and exposure time combination for a constant energy density on temperature gradient

For constant energy density, Fig. 6 shows that decreasing power and increasing exposure results in lowering the highest temperature in the melt pool which is consistent with the findings of Ref 24, 25). Studying the effect of decreasing power individually Manvatkar et al. (Ref 24) reported decreased melt-pool size and increased cooling rate. Since the current study varied both power and exposure proportionally for keeping energy density constant, reducing power did not affect melt-pool size (see Fig. 2) while cooling rate decreased (see Fig. 3). Figure 7 shows a decrease in temperature gradient between the top and 250 µm depth across a melt pool, as illustrated by the slope of the line equations which is consistent with the findings of Vasinonta et al. (Ref 7), reporting lower thermal gradients for slower scanning.

**Fig. 8:**
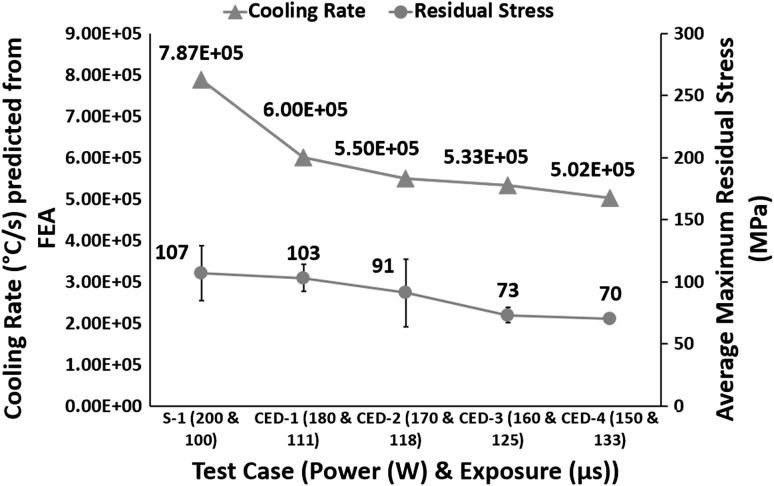
Effect of varying power and exposure time combination for a constant energy density on cooling rate and residual stress

For constant energy density, Fig. 8 shows a decreasing trend in residual stress and cooling rates with decreasing power and increasing exposure. Figure 8 shows test case S-1, manufactured with optimum combination of power (200 W) and exposure (100 µs), resulted in 107 MPa residual stress. CED-1 resulted in 3.7% reduction in residual stress compared with S-1. CED-2 resulted in 15% reduction in residual stress compared with S-1. CED-3 resulted in 19.8% decrease in residual stress compared with CED-2 and 31.8% compared with S-1. CED-4 resulted in 4.1% decrease in residual stress compared with CED-3 and 34.6% compared with S-1.

The decreasing trend in cooling rate is consistent with the work by Manvatkar et al. (Ref 24), reporting decreased cooling rate for slower scanning. This decrease in cooling rate leads to a decrease in residual stress in samples made with lower power and higher exposure. Brückner et al. (Ref 26) reported slower scan speed led to reducing residual stress in a single track. Therefore, it is valid to suggest that maintaining the energy density constant, the trend in cooling rate and residual stress follows the same trend as when the effect of exposure time on residual stress is studied individually. The correlation of cooling rate and residual stress with power is the opposite of when power is varied individually. Decreased power and increased exposure combination leads to lower temperature gradients (see Fig. 7) and lower cooling rates (see Fig. 8). Thus, according to the temperature gradient mechanism (Ref 33) and cool-down phase model (Ref 16, 33), decreasing power and increasing exposure keeping energy density constant should lead to a decrease in residual stress.

#### Effect of Laser Power and Exposure with Constant Energy Density on Mechanical Properties

**Fig. 9:**
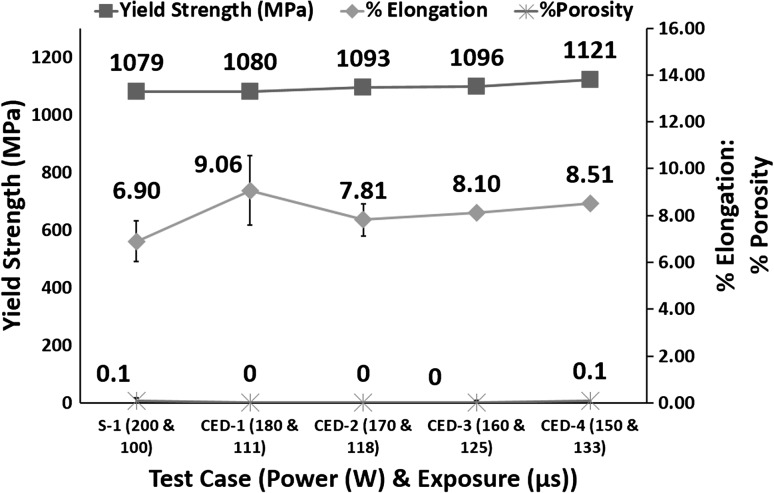
Effect of varying power and exposure time combination for a constant energy density on % porosity, % elongation and yield strength

Figure 9 shows decreasing power and increasing exposure leads to a slight increase in yield strength, while there is a considerable improvement in % elongation of SLM samples while % porosity remains consistent.

Figure 3 shows that for constant energy density, decreasing power and increasing exposure leads to reduction in cooling rates. According to effective slip length and dislocation movement theories (Ref 34, 35), decreasing power and increasing exposure should lead to a decrease in yield strength, as it decreases the cooling rate. According to Leuders et al. (Ref 36), process-induced porosity acts as a stress concentrator and leads to a reduction in mechanical properties. Figure 9 shows that for test cases CED-1 to CED-3 there is no porosity which might be the reason for 1.6% increase in yield strength of CED-3 compared with S-1. CED-4 shows 0.1% porosity, similar to S-1, and much lower cooling rate, but resulted in 3.9% increase in yield strength compared to S-1. Varying combinations of power and exposure keeping energy density constant affect the yield strength of the samples in the range of 1-3%.

Figure 9 shows an increasing trend in ductility with decreasing power and increasing exposure keeping energy density constant. The sudden increase in the elongation of CED-1 is not clear as it has cooling rates higher than that of test cases CED-2 to CED-4. Overall Fig. 9 shows that lower power and higher exposure combinations leads to an increase in elongation. According to effective slip length and dislocation movement theories (Ref 34, 35), ductility increases with increasing cooling rate up to a certain point (around 500-600 $\frac{{{^\circ }{\text{C}}}}{\text{s}}$), and beyond this point of maximum ductility, it decreases sharply with a further increase in the cooling rate. This intermediate optimum cooling rate for maximum ductility (Ref 34, 35) is much lower than SLM cooling rates. The SLM cooling rate decreases with decreasing power and increasing exposure which leads to an increase in ductility as the cooling rate is moving toward the intermediate optimum cooling rate for maximized ductility.

**Fig. 10:**
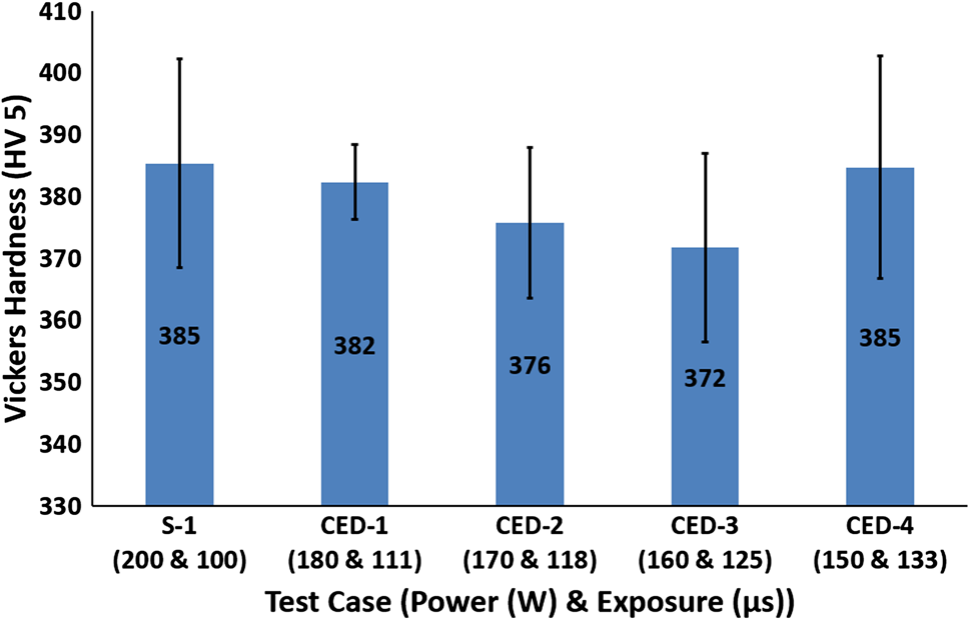
Effect of varying power and exposure time combination for a constant energy density on Vickers hardness

All the test cases had a totally martensitic microstructure (see Fig. 3 for cooling rates); therefore, Fig. 10 shows no major variation in Vickers hardness.

### Effect of Layer Thickness

FEA simulation with different layer thicknesses was used to estimate the effect of layer thickness on cooling rates and temperature gradients.

**Fig. 11:**
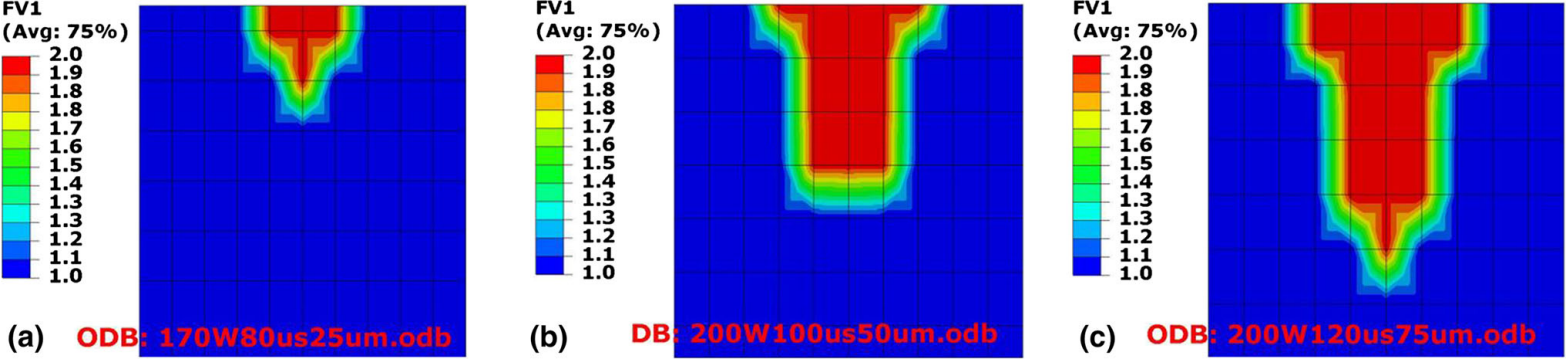
Effect of layer thickness on melt-pool dimensions. (a) Test case LT-1 (25 µm layer thickness). (b) Test case LT-2 (50 µm layer thickness). (c) Test case LT-3 (75 µm layer thickness)

Figure 11 shows a direct relationship between layer thickness and melt-pool size.

**Fig. 12:**
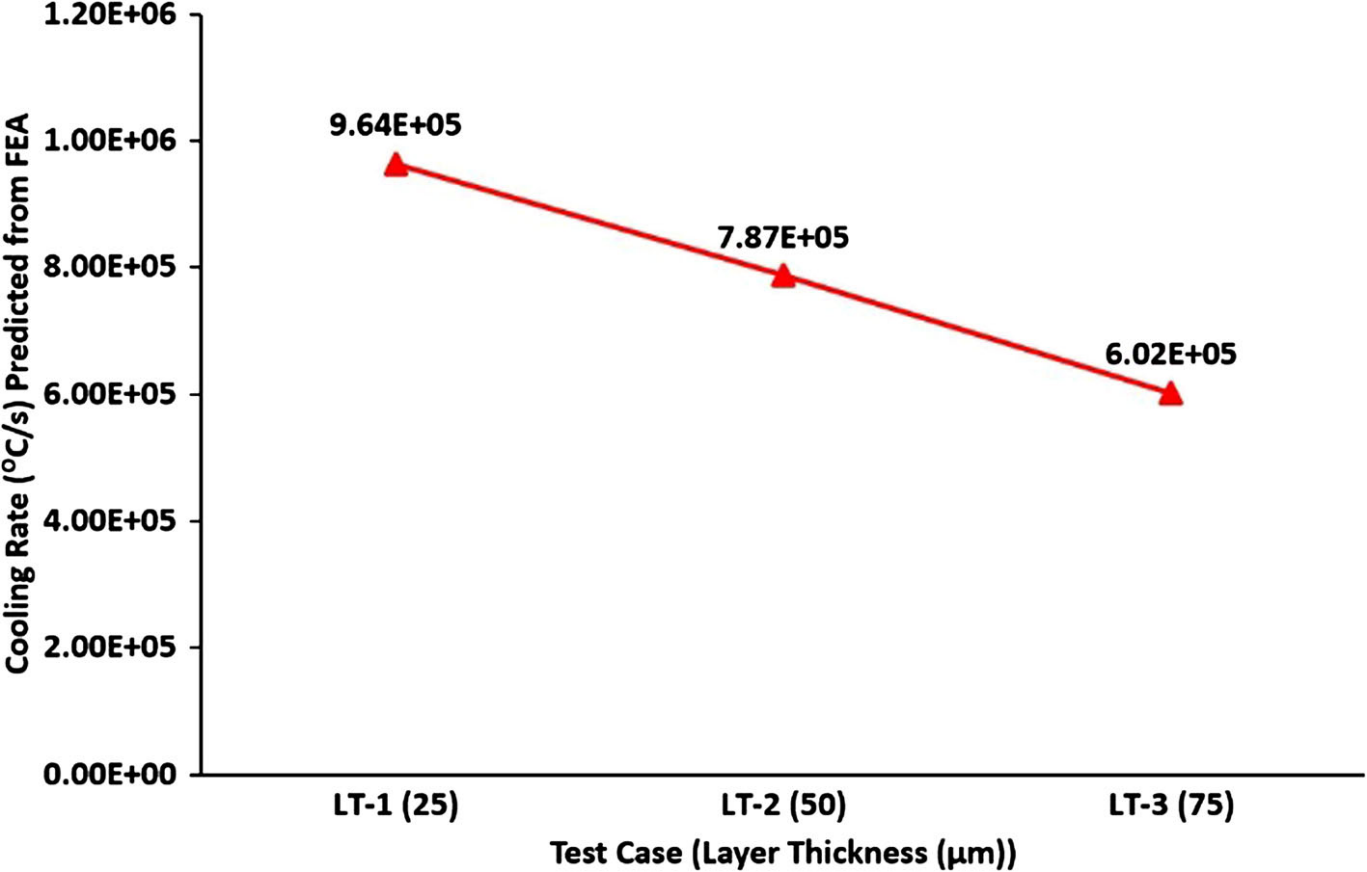
Effect of layer thickness on cooling rates

A larger melt pool contains a higher volume of processed material that will cool at reduced cooling rate; therefore, Fig. 12 shows an inverse relationship between layer thickness and cooling rates.

#### Effect of Layer Thickness on Porosity and Microstructure

Increasing layer thickness resulted in an increase in porosity even though the parameters were optimized for each layer thickness.

**Fig. 13:**
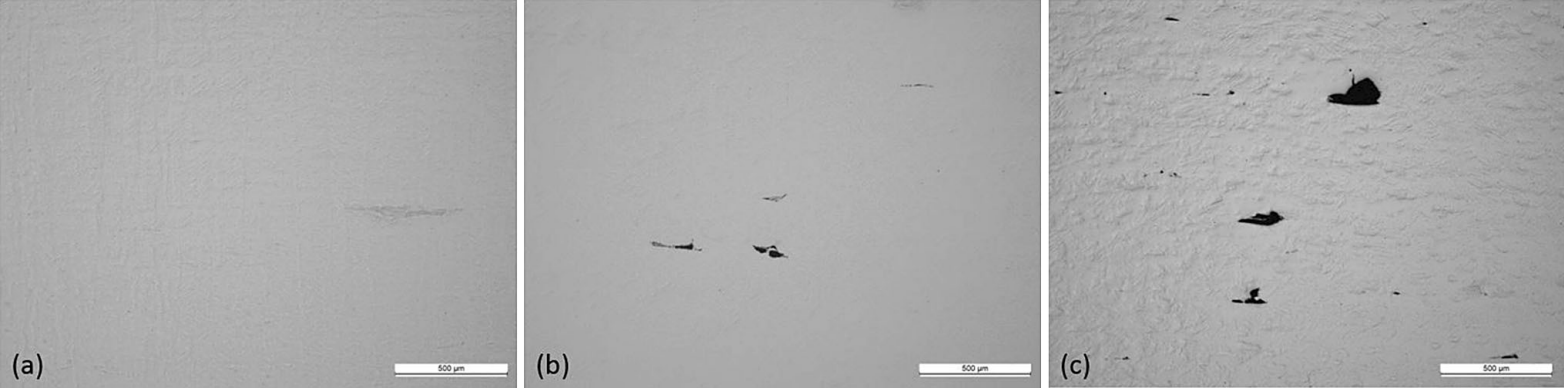
Interlayer defects (a) LT-1, (b) LT-2 and (c) LT-3

**Fig. 14:**
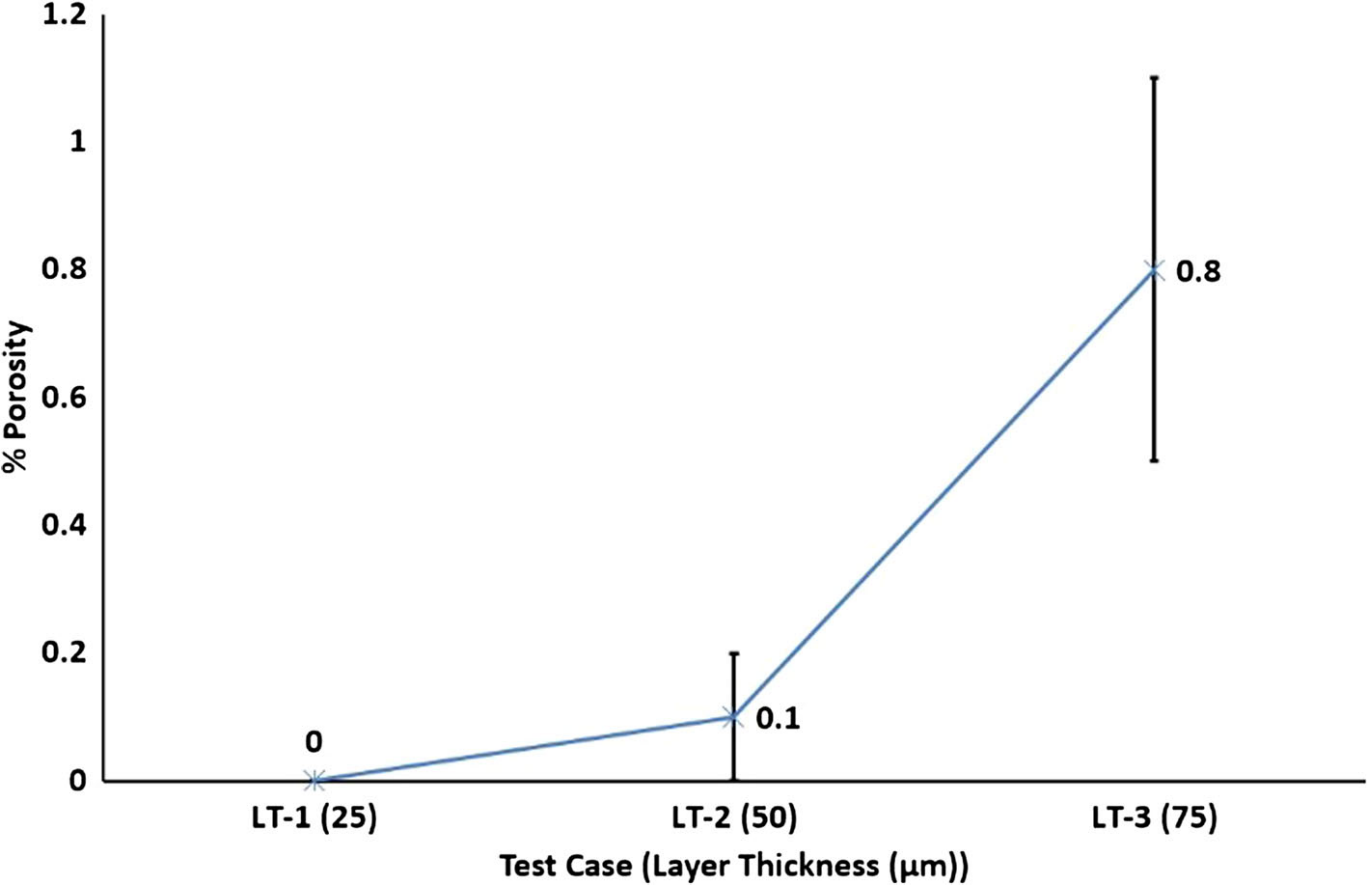
Effect of layer thickness on % porosity

Figure 13 shows an increase in inter layer defects which led to an increase in % porosity with increasing layer thickness. Figure 14 shows LT-1 resulted in 0% porosity, increasing to 0.1% for LT-2 and 0.8% for LT-3.

**Fig. 15:**
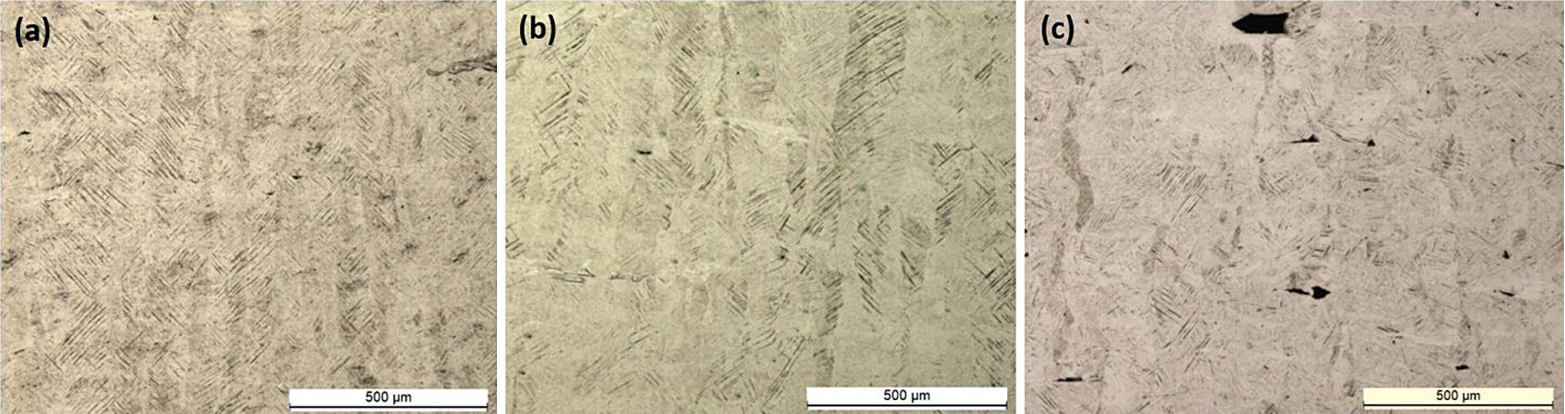
Martensitic $\alpha^{\prime}$ laths in prior β columnar grains. (a) Test case LT-1, 25 µm layer thickness. (b) Test case LT-2, 50 µm layer thickness. (c) Test case LT-3, 75 µm layer thickness

Figure 12 shows increasing layer thickness led to reduced cooling rates but still much higher than the cooling rate required for a fully martensitic microstructure in Ti6Al4V. Ahmed et al. (Ref 32) reported cooling rates higher than 410 $\frac{{{^\circ }{\text{C}}}}{\text{s}}$ leads to fully martensitic microstructure for Ti6Al4V. Therefore, irrespective of the layer thickness all test cases resulted in fully martensitic microstructure with martensitic $\alpha^{\prime}$ laths growing inside prior columnar β grains as shown in Fig. 15.

#### Effect of Layer Thickness on Residual Stress

**Fig. 16:**
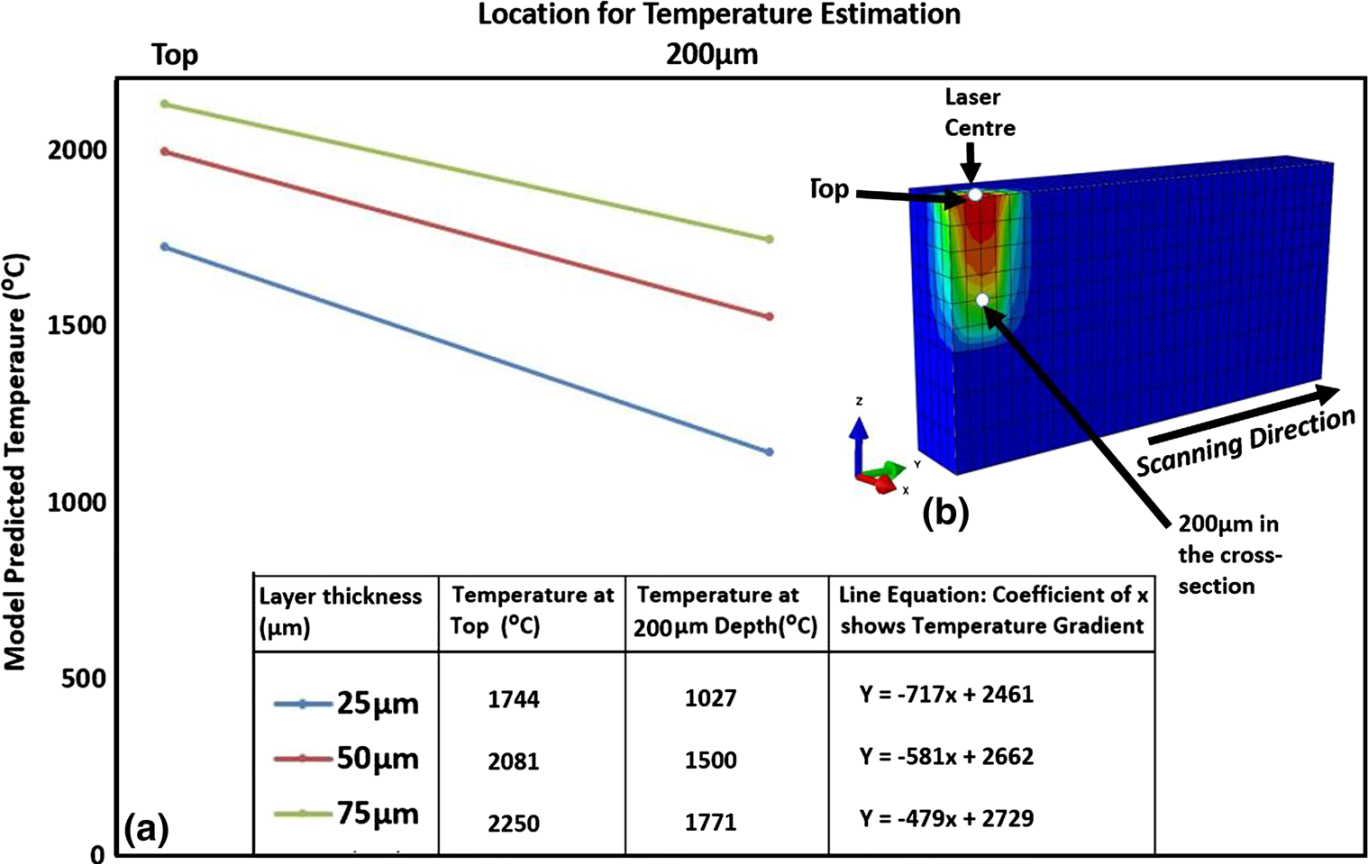
Effect of layer thickness on temperature gradient

Figure 16 shows that increasing layer thickness results in increasing the peak temperature in the melt pool. Test case LT-1 was built with 170 W and 80 µs, resulting in an energy density of 104.62 $\frac{\text{J}}{{{\text{mm}}^{3} }}$. LT-2 was built with 200 W and 100 µs, resulting in an energy density of 76.92 $\frac{\text{J}}{{{\text{mm}}^{3} }}$. LT-3 was built with 200 W and 120 µs, resulting in an energy density of 61.54 $\frac{\text{J}}{{{\text{mm}}^{3} }}$. This shows that the required energy density for fully dense parts decreased with increasing layer thickness. The only probable explanation for this behavior is that increasing powder layer thickness hinders the conduction of heat away to the substrate, and thus, more energy is retained in the powder. This leads to higher peak temperatures (see Fig. 16) and larger melt-pool size (see Fig. 11). Another important feature from Fig. 16 is the decrease in temperature gradient with increasing layer thickness between the top and 200 µm depth across a melt pool, as illustrated by the slope of the line equations. Thus, according to the temperature gradient mechanism (Ref 33) and cool-down phase model (Ref 16, 33), increasing layer thickness should lead to a decrease in residual stress.

**Fig. 17:**
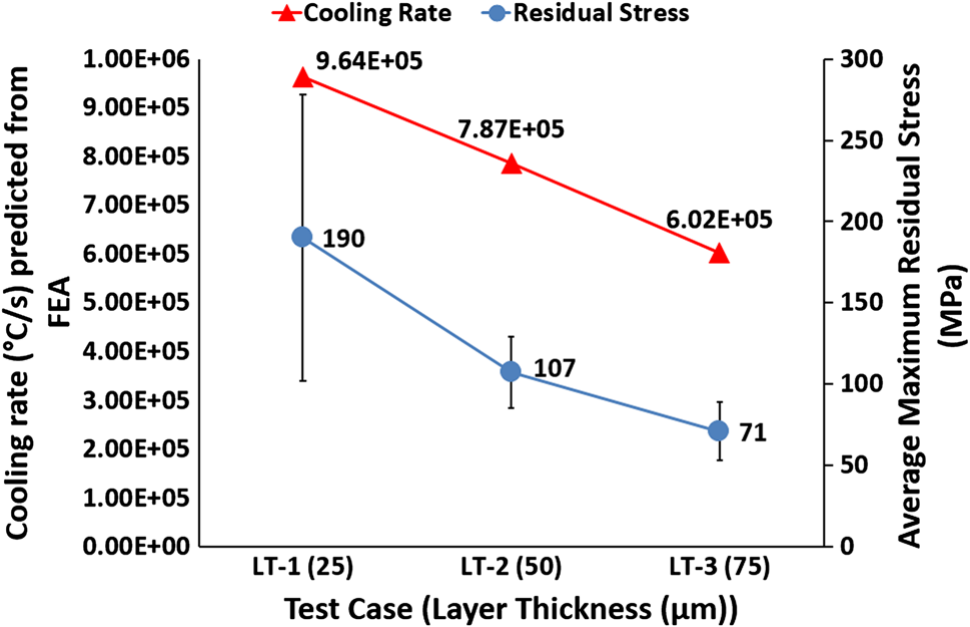
Effect of layer thickness on cooling rate and residual stress

Figure 17 shows an inverse relation between residual stress and layer thickness. Test case LT-1 resulted in 190 MPa residual stress. LT-2 showed a decrease of 43.7% in residual stress, compared to LT-1. LT-3 resulted in a further decrease of 27.1% compared to LT-2 and 58.9% compared to LT-1. A decrease in residual stress with increasing layer thickness is consistent with the findings of Zaeh et al. (Ref 14), reporting a reduction in deformation of cantilever specimens with increasing layer thickness. Kruth et al. (Ref 18) also reported a decreasing trend in the deformation of bridge-shaped specimens with increasing layer thickness. Van Belle et al. (Ref 12) also reported a reduction in deformation of thin plates onto which powder layers were deposited with increasing layer thickness. Figure 16 shows a decrease in thermal gradient, and Fig. 17 shows a reduction in cooling rates with increasing layer thickness; therefore, according to the temperature gradient mechanism (Ref 33) and cool-down phase model (Ref 16, 33), increasing layer thickness leads to a decrease in residual stress.

#### Effect of Layer Thickness on Mechanical Properties

**Fig. 18:**
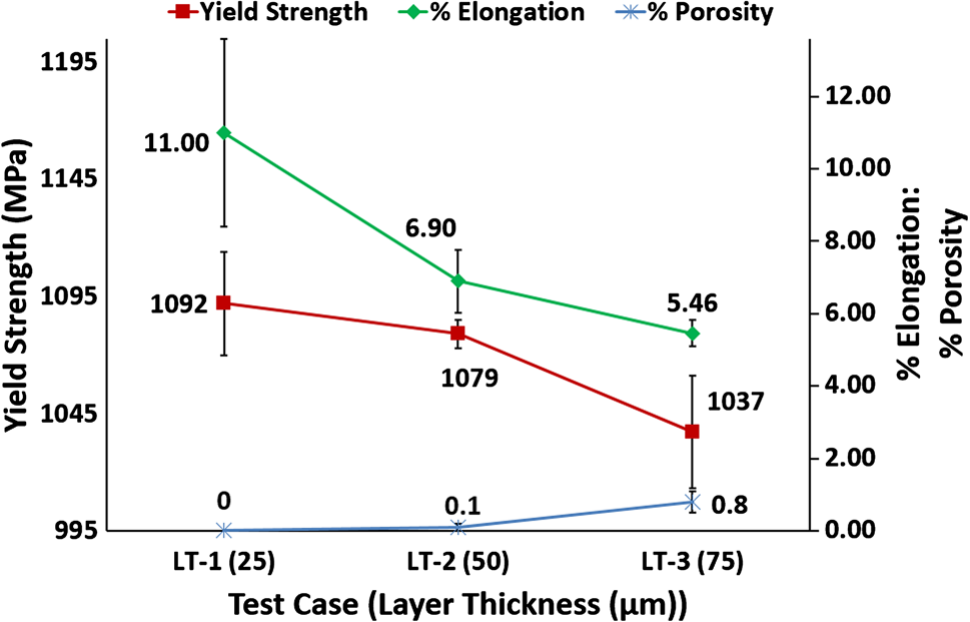
Relationship between layer thickness, % porosity, % elongation and yield strength

Figure 18 shows a decreasing trend in % elongation and yield strength with increasing layer thickness, while % porosity increases.

LT-1 showed a yield strength of 1092 MPa. LT-2 resulted in a decrease of 1.2% in yield strength, compared to LT-1. LT-3 resulted in a further decrease of 3.9% compared to LT-2 and 5% compared to LT-1. It is therefore clear from the results shown in Fig. 18 that increasing layer thickness resulted in under 5% reduction in yield strength.

Figure 12 shows increasing layer thickness led to a reduction in cooling rates. For lamellar microstructure, the mechanical properties are greatly affected by the α colony size (Ref 34, 35). Colony size determines the effective slip length and is inversely proportional to the cooling rate from the β phase field. According to Ref 34, 35), yield strength is inversely proportional to slip length and yield strength grows exponentially with cooling rate, over 1000 $\frac{{{^\circ }{\text{C}}}}{ \hbox{min} }$ (air cooling). Manikandakumar et al. (Ref 37) reported mechanical properties of SLM Ti6Al4V parts depend on the α colony and α lath size. The α lath and α colony sizes are equal to single martensitic $\alpha^{\prime}$ laths for a martensitic microstructure. The movement of dislocations is restricted due to the smaller α colony sizes in martensitic microstructures for SLM Ti6Al4V, which leads to limited plastic deformation in SLM Ti6Al4V components. Limited plastic deformation of SLM parts leads to higher yield strength and UTS. According to effective slip length and dislocation movement theories (Ref 34, 35), increasing layer thickness should lead to a decrease in yield strength as increased layer thickness means slower cooling rate and thus lower yield strength. According to Leuders et al. (Ref 36), specimens can fail prematurely due to process-induced porosity acting as stress concentrators. Figure 18 shows that increasing layer thickness leads to an increase in porosity. Therefore, the increase in inter layer porosity with increasing layer thickness is another factor contributing to the reduction in yield strength with increasing layer thickness.

Figure 18 shows inverse relationship between % elongation and layer thickness. Test case LT-1 resulted in 11% elongation. LT-2 resulted in a decrease of 37.3% in elongation, compared to LT-1. LT-3 resulted in a further decrease of 20.9% compared to LT-2 and 50.2% compared to LT-1. It is therefore clear from the results shown in Fig. 18 that increasing the layer thickness resulted in a significant reduction in the elongation of the samples.

The relationship between cooling rates and ductility is more complex (Ref 34, 35). Decrease in slip length leads to an increase in ductility (Ref 34, 35). Ductility increases with increasing cooling rate up to a certain point (500-600 $\frac{{{^\circ }{\text{C}}}}{\text{s}}$), and beyond this point of maximum ductility, it decreases sharply with further increase in the cooling rate (Ref 34, 35). The intermediate cooling rate resulting in maximum ductility is much lower than the SLM cooling rates. The cooling rate decreases with increasing layer thickness which should lead to an increase in ductility as the cooling rate is moving toward the intermediate optimum cooling rate for maximized ductility. Since the ductility is decreasing despite the cooling rates moving toward the optimum, the only explanation for this decrease can be attributed to the increase in inter layer porosity with increasing layer thickness. Therefore, it is valid to say that porosity defects act as stress concentrators, which leads to premature failure of tensile specimens and thus results in the deterioration of mechanical properties.

**Fig. 19:**
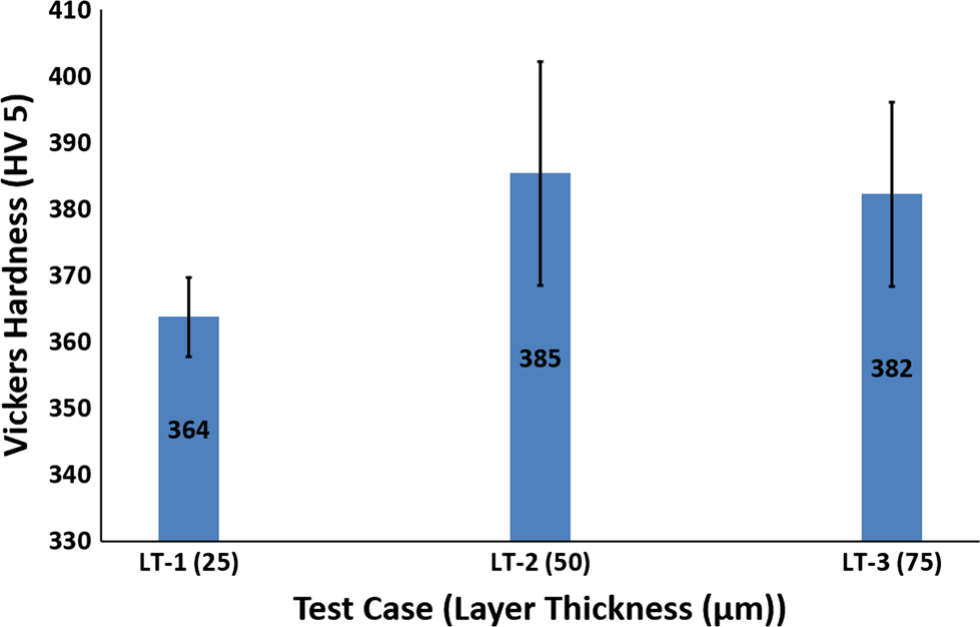
Effect of layer thickness on Vickers hardness

All the test cases had a totally martensitic microstructure (see Fig. 12 for cooling rates); therefore, Fig. 19 shows no major variation in Vickers hardness.

## Conclusions

Keeping energy density constant (optimum energy density determined from parameter optimization), the effect of varying power and exposure combination on residual stress and mechanical properties was investigated. For constant energy density, the FEA model predicted a direct relationship between power, cooling rates and temperature gradients and an inverse relationship between exposure, cooling rates and temperature gradients. All samples resulted in fully martensitic microstructure with prior columnar beta grains irrespective of the power and exposure combination as the cooling rates were much higher than 410 $\frac{{{^\circ }{\text{C}}}}{\text{s}}$. For constant energy density, lower power and higher exposure combination resulted in lower residual stress in SLM Ti6Al4V components. 150 W and 133 µs combination resulted in lowest residual stress due to lower cooling rate and lower temperature gradient. For constant energy density, the yield strength did not show any considerable variation with power and exposure. The % elongation showed an increasing trend with decreasing power and increasing exposure resulting from increase in α lath size due to a decrease in cooling rate.

Three different layer thicknesses (25, 50 and 75 µm) were investigated to understand the effect on residual stress, microstructure and mechanical properties of SLM Ti6Al4V components. FEA model predicted an inverse relationship between layer thickness and cooling rates because of the increase in melt-pool size with layer thickness. Layer thickness and temperature gradients are also inversely related. All samples resulted in fully martensitic microstructure with prior columnar beta grains irrespective of the layer thickness as the cooling rates were much higher than 410 $\frac{{{^\circ }{\text{C}}}}{\text{s}}$. Layer thickness showed an inverse relationship with experimentally measured residual stress. Layer thickness of 75 µm resulted in the lowest residual stress due to lower cooling rate and lower temperature gradients. Yield strength and elongation showed an inverse relationship with layer thickness. Layer thickness of 25 µm resulted in the highest yield strength and elongation values for SLM Ti6Al4V components as the samples had no visible interlayer defects.
